# Quantitative profiling of lifespan-dependent cell-cell communication potential reveals dynamic ligand-receptor network shifts across mouse tissues

**DOI:** 10.1371/journal.pone.0345045

**Published:** 2026-03-20

**Authors:** Boyong Wei, Evan P. Troendle, David A. Simpson, Alan W. Stitt

**Affiliations:** Wellcome-Wolfson Institute for Experimental Medicine, School of Medicine, Dentistry and Biomedical Sciences, Queen’s University Belfast, Belfast, United Kingdom; ShanghaiTech University, CHINA

## Abstract

Cell-to-cell communication (CCC) is a tightly regulated process essential for tissue development and homeostasis, but can become dysregulated during ageing. While CCC is inherently complex and remains incompletely characterised, advances in single-cell RNA sequencing (scRNA-seq) have enabled large-scale, unbiased inference of intercellular interactions which offers broad-spectrum information that complements traditional protein-based assays. Unlike these targeted assays, transcriptomic approaches enable systematic inference and exploration of both known and potentially novel ligand-receptor (LR) interactions. In this study, we applied LIgand-receptor ANalysis frAmework (LIANA), which integrates multiple inference methods to derive consensus CCC predictions, to scRNA-seq data for four mouse organs (liver, lung, heart, and kidney), spanning key life stages: post-natal development, adulthood and ageing. Our analysis revealed dynamic, organ-specific CCC patterns characterised by both gains and losses of LR interactions over time, reflecting lifespan-dependent shifts in transcriptome-inferred intercellular communication potential. To quantify these shifts, we developed a two-phase comparative framework and introduced the Shrink and Expand (SE) score to capture directional changes in inferred LR interaction sets between any two biological states. Applying this framework generated a curated dataset of LR pairs and their predicted changes, capturing the repertoire of putative interactions across organs and states and enabling robust, interpretable comparisons of organ-specific and coinciding patterns of change across multiple organs. For instance, CD44 and ITGB1 were found to undergo highly dynamic changes across timepoints and organs, suggesting that they may act as central nodes in predicted age-dependent communication changes. This generalisable approach supports quantitative comparisons of inferred CCC across diverse states, including development, ageing, disease, or treatment conditions, and provides a resource for prioritising candidate interactions for drug target discovery for further experimental validation while exploring context-specific shifts in predicted intercellular communication.

## Introduction

Cell-to-cell communication (CCC) is fundamental to multicellular organisms, enabling individual cells to orchestrate activities that preserve tissue architecture and organ-level function throughout development, homeostasis, and repair [1,2]. CCC is considered highly complex as it is mediated through diverse mechanisms, including direct membrane-bound interactions between adjacent cells (e.g., juxtracrine signalling [3]) and diffusible signalling via secreted molecules acting in autocrine, paracrine, or endocrine pathways [4]. Although many ligand-receptor (LR) interactions have been experimentally validated, a considerable proportion remains completely uncharacterised, reflecting the challenge to resolve intercellular communications networks [5], particularly within complex and heterogeneous tissue environments.

This complexity becomes especially evident during ageing, a process marked by altered CCC that contributes to progressive functional decline [6]. A notable feature of ageing is the senescence-associated secretory phenotype (SASP), wherein senescent cells secrete pro-inflammatory factors that may propagate senescence and dysregulated signalling in neighbouring cells, potentially disrupting tissue homeostasis [7].

Traditional approaches for studying CCC, such as targeted protein-based assays and three-dimensional cell culture models, have provided valuable mechanistic insights into intercellular signalling [8–10]. However, these approaches are inherently constrained by their focus on predefined LR pairs and limited scalability, restricting their capacity to comprehensively capture CCC dynamics within complex, heterogeneous tissues [11].

Recent advances in high-throughput transcriptomics, particularly single-cell RNA sequencing (scRNA-seq), have substantially expanded our capacity to profile gene expression at single-cell resolution [11,12]. This enables the inference of putative ligand–receptor interactions across diverse cell types and tissue contexts, providing a powerful, albeit indirect, approach to exploring intercellular communication networks. It should be noted that transcriptomic profiling primarily captures peptide-based ligands, and therefore, non-peptide mediators of intercellular communication (e.g., steroid hormones, metabolites, ions) [13] are not represented in this framework. While this represents a known limitation, transcriptomics nonetheless provides a scalable and systematic approach to infer peptide-mediated signalling at single-cell resolution.

To capitalise on vast scRNA-seq data, computational frameworks have been developed to infer CCC from transcriptomic profiles, integrating multiple methods to predict intercellular interactions and uncovering novel communication networks. Among these, LIgand-receptor ANalysis framework (LIANA) consolidates outputs from several inference algorithms to provide a consensus, high-confidence readout of CCC interactions [14]. While LIANA systematically scores LR interactions present in a given dataset, it does not explicitly quantify directional changes or network-level expansions and contractions across states or stages.

Here, we applied LIANA to systematically explore predicted age-associated CCC changes across four mouse organs: the liver, lung, heart, and kidney, which were selected for their distinct physiological roles, well-documented involvement in age-related functional decline [15–18], and representation in the *Tabula Muris Senis* dataset, which spans postnatal development, adulthood, and ageing [19]. Using a two-phase comparative strategy, we analysed consecutive age groups to infer potential shifts in intercellular signalling, with an emphasis on organ-specific patterns. To capture directional shifts in LR interactions, we introduce the Shrink and Expand (SE) score, a novel metric that quantifies predicted net gains or losses of communication pairs between biological states. Unlike traditional symmetric similarity measures (e.g., the Jaccard Index [20]), the SE score separately accounts for expansions and shrinkages in communication, providing a measure for putative directional changes between communication states. This score, bounded between −1 and 1, allows comparison of potential CCC remodelling across tissues and stages, offering into how inferred communication patterns may evolve during ageing.

Together, this integrated analytical framework offers a powerful toolset to explore temporal and organ-specific patterns of cellular communication, advancing our understanding and ability to generate hypotheses surrounding age-related changes predicted from transcriptomic inference.

## Methods

### Data source

**Fig 1:**
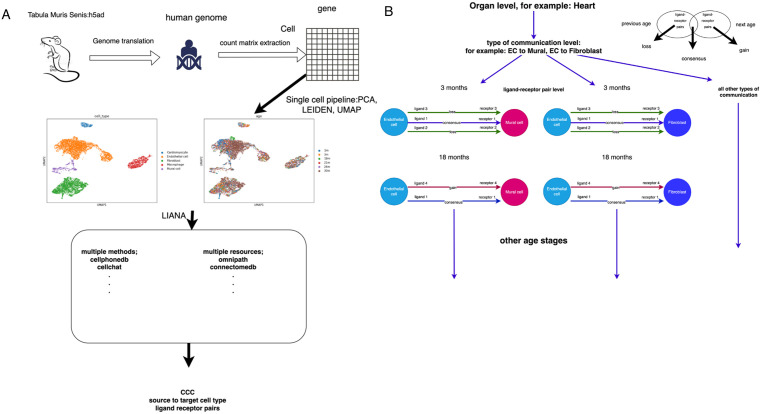
(A). Summary of the workflow used to generate cell to cell communication results from single cell RNA sequence h5ad data from the Tabula Muris Senis dataset. (B): Structure of CCC analysis between ages. The main strategy is two-phase comparison where all ligand-receptor pairs are categorised according to their presence in two-phase ages.

scRNA-seq data were obtained from the *Tabula Muris Senis* (TMS) dataset (Fig 1A) [19]. Samples generated using the 10x Genomics 3’ v2 protocol from four organs—liver, lung, heart, and kidney—were selected for this study. Raw expression count matrices were extracted from the.h5ad files downloaded directly from the TMS website (https://tabula-muris-senis.ds.czbiohub.org/).

The TMS repository contains data from mice aged 1, 3, 18, 21, 24 and 30 months. For our time-adjacent lifespan comparisons we used the consecutive age intervals 1–3, 3–18, 18–21 and 21–30 months. These contrasts were defined by the directly neighbouring ages available in the dataset, ensuring that each interval represented the smallest possible progression suitable for subsequent comparative analyses of inferred communication potential.

Although 24-month samples are publicly available, we excluded them from these pairwise analyses because the captured cell-type diversity at this age was markedly and unevenly reduced across several organs. This was particularly evident in the kidney, where only a limited set of immune populations were recovered. These imbalances are evident in Table 1 in S1 Table, which reports full cell-type distributions across all ages. Including these data would likely have confounded interpretation by conflating age-associated transcriptomic patterns with dataset-specific sampling artefacts (e.g., dissociation or sequencing biases).

### Data import and cross-species translation

The extracted matrices were imported into Scanpy (version 1.9.6; [21]) to create initial objects for downstream analysis. To simulate human physiological conditions without introducing single nucleotide polymorphism (SNP) variability [22], the mouse genome annotations were translated into the human genome using Mousipy (version 0.1.3; https://pypi.org/project/mousipy/; [23]). If there are multiple different human orthologs, the gene’s expression counts are added to all its orthologs. This translation ensured cross-species compatibility while preserving the transcriptomic context, as depicted in Fig 1A.

All gene symbols reported in the text and figures are official human gene symbols as defined by the HUGO Gene Nomenclature Committee (HGNC; [24]). Mousipy uses Mouse Genome Informatics (MGI; [25]) data internally for mouse–human orthology but outputs only HGNC symbols.

### Single cell data normalisation, dimensionality reduction, and clustering analysis

Cell populations with fewer than 200 genes expressed were removed to exclude low-quality cells and empty droplets, ensuring sufficient transcriptomic complexity for downstream analyses. Additionally, genes detected in fewer than three cells were removed to eliminate minimally expressed genes, and cells with mitochondrial gene counts exceeding 10% were excluded to minimize potential contamination by damaged or dying cells. The remaining data were normalised to 10,000 unique molecular identifiers (UMIs) per cell and subsequently log-transformed (log1p). To identify highly variable genes, the ‘pp.highly_variable_genes’ function in Scanpy was applied [21], using parameters set according to the official Scanpy tutorial (min_mean = 0.0125, max_mean = 3, min_disp = 0.5).

Principal component analysis (PCA) was then performed to reduce the dimensionality of the dataset, using the default settings in Scanpy. For visualisation, Uniform Manifold Approximation and Projection (UMAP) was applied with 30 principal components and a neighbour parameter of 10 [26–28]. Finally, clustering was performed using the Leiden algorithm [26] (resolution = 0.25) based on the UMAP results. Clusters containing fewer than 150 cells were excluded from further analysis to balance the inclusion of rare cell types with the need for sufficient statistical power.

### Cell type annotation

Cell type annotation was accomplished using a marker-based approach. Differential gene expression between clusters was assessed via a T-test using the ‘tl.rank_genes_groups’ function in Scanpy. The top 20 differentially expressed genes for each cluster, ranked by log-fold change, were selected for further analysis. These candidate marker genes were cross-referenced with the Human Protein Atlas (https://www.proteinatlas.org/). The specific annotations and marker genes used for cell type identification are provided in Table 1 in S1 Table.

### Cell-to-cell communication inference

All CCC inferences were generated using LIANA version 0.1.9 [14] (Fig 1A). The analysis followed the parameters and platform settings outlined in the official LIANA tutorial (https://liana-py.readthedocs.io/en/latest/notebooks/basic_usage.html). The ‘rank_aggregate’ function was applied with the following parameters grouping by cell type (groupby=’cell_type’), using the consensus resource (resource_name = ’consensus’), setting a minimum expression proportion of 0.1 (expr_prop = 0.1), requiring at least 5 cells per cell type (min_cells = 5), with the base of the natural logarithm set to e (base = 2.718281828459045), using robust rank aggregation as the aggregation method (aggregate_method = ’rra’), not returning all LR pairs (return_all_lrs = False), no additional consensus options (consensus_opts = None), analysis performed on non-raw data (use_raw = False), no specific data layer specified (layer = None), differential expression calculated via t-test (de_method = ’t-test’), verbose output disabled (verbose = False), 1000 permutations for significance testing (n_perms = 1000), a fixed random seed for reproducibility (seed = 1337), no alternative resource specified (resource = None), and results returned without modifying the input object (inplace = False).

The ‘rank_aggregate’ function was used to aggregate communication scores and acquire the most reliable results. A double p-value filtering strategy was implemented, whereby only interactions with p-values below 0.05 in both CellPhoneDB [29] and CellChat [12] methods were considered for further analysis. This stringent filtering ensured the inclusion of only the most robust and significant interactions in our study.

Note that in this study, in line with LIANA and its underlying interaction resources (e.g., OmniPath [30], CellPhoneDB [29]), we use the term “ligand–receptor” (LR) pair as a convenient shorthand to refer broadly to intercellular protein–protein interaction pairs. These include not only classical secreted ligand → transmembrane receptor signalling events, but also other experimentally supported interaction types, such as receptor–receptor and receptor–intracellular effector associations. Because these databases aggregate interactions from hundreds of published studies, the resulting compendium reflects a broad set of candidate molecular communication pathways, rather than being restricted to canonical mechanisms.

LIANA performs inference agnostically across all supplied cells, generating predicted LR interactions for every possible pair of cells or clusters [14]. All inferred pairs represent statistically inferred communication potential, derived from transcriptomic co-expression and prior interaction knowledge, rather than experimentally validated signaling between distinct cells.

### Generation of shrink and expand (SE) scores

To capture directional changes in predicted CCC, we introduce the *Shrink and Expand (SE) score*, a directional set similarity metric that quantifies predicted changes in LR co-expression patterns between cell types across two biological states. Unlike symmetric indices such as the Jaccard index [20], SE separately captures expansions (gains of new predicted LR pairs) and shrinkages (potential losses of predicted LR pairs), providing a measure of potential directional changes in intercellular communication inferred from transcriptomic data. While conceptually related to asymmetric set similarity metrics such as the Tversky index [31], the SE score is distinct in its formulation: it explicitly decomposes communication changes into directional components (gains vs. losses) and expresses their difference on a bounded scale. This allows straightforward interpretation of relative gains and losses of LR co-expression, making it particularly well suited for CCC analysis across developmental stages, conditions, or treatments.

Let A and B denote the two putatively interacting cell types, potentially involved in CCC, where A may equal B, defining a communication inference type $t_{A↔B}$. For two biological states (e.g., consecutive developmental stages, disease vs. healthy, treated vs. untreated, etc.) labelled s=1 (reference) and s=2 (comparator), we define the sets of predicted LR pairs for communication inference type $t_{A↔B}$ as $L_{t_{A↔B}}^{\left(\text{1}\right)}$ and $L_{t_{A↔B}}^{\left(\text{2}\right)}$, with cardinalities $\left|L_{t_{A↔B}}^{\left(\text{1}\right)}\right|$ and $\left|L_{t_{A↔B}}^{\left(\text{2}\right)}\right|$, respectively, representing the number of LR interactions predicted in each state. Each transcriptome-inferred LR pair, as predicted within a given communication inference type $t_{A↔B}$, constitutes a ‘CCC inference unit’, which will later be classified to assess directional changes.

The intersection of these sets, denoted:


$$I_{t_{A↔B}}\;=L_{t_{A↔B}}^{\left(\text{1}\right)}\cap L_{t_{A↔B}}^{\left(\text{2}\right)}$$

represents the set of conserved LR pairs between the two states, with cardinality $\left|I_{t_{A↔B}}\right|=\left|L_{t_{A↔B}}^{\left(\text{1}\right)}\cap L_{t_{A↔B}}^{\left(\text{2}\right)}\right|$.

To quantify directional change in CCC potential, we next compute the intersection ratios, which each represent the proportion of conserved interactions relative to each state:


$$r_{t_{A↔B}}^{\left(\text{1}\right)}=\frac{\left|L_{t_{A↔B}}^{\left(\text{1}\right)}\cap L_{t_{A↔B}}^{\left(\text{2}\right)}\right|}{\left|L_{t_{A↔B}}^{\left(\text{1}\right)}\right|}\text{ and }r_{t_{A↔B}}^{\left(\text{2}\right)}=\frac{\left|L_{t_{A↔B}}^{\left(\text{1}\right)}\cap L_{t_{A↔B}}^{\left(\text{2}\right)}\right|}{\left|L_{t_{A↔B}}^{\left(\text{2}\right)}\right|}$$

Here, for communication type $t_{A↔B}$, $r_{t_{A↔B}}^{\left(\text{1}\right)}$ captures the proportion of putative state 1 (reference) interactions retained in state 2 (comparator), whereas $r_{t_{A↔B}}^{\left(\text{2}\right)}$ measures the proportion of putative state 2 interactions that were already deemed present in state 1.

The SE score for communication type $t_{A↔B}$ is then defined as the difference between these two ratios:


$$SE_{t_{A↔B}}=r_{t_{A↔B}}^{\left(\text{2}\right)}-r_{t_{A↔B}}^{\left(\text{1}\right)}=\left|I_{t_{A↔B}}\right|\left(\frac{\text{1}}{\left|L_{t_{A↔B}}^{\left(\text{2}\right)}\right|}-\frac{\text{1}}{\left|L_{t_{A↔B}}^{\left(\text{1}\right)}\right|}\right)$$

This score is bounded between –1 and 1, where positive values (SE > 0) indicate net potential expansion, meaning the comparator state (2) contains new LR interactions not inferred in the reference (1). Negative values (SE < 0) denote net shrinkage, reflecting the loss of LR interactions that were inferred in the reference (1) but absent in the comparator (2). A score of zero (SE = 0) reflects a net balance between gains and losses of LR pairs. This does not necessarily imply that the communication network itself is unchanged, but rather that predicted expansions and contractions of CCC potential are proportionally balanced.

This directional, normalised score enables direct comparison of inferred CCC dynamics across different organs, conditions, and timepoints, regardless of variation in the total number of predicted LR pairs. Therefore, the SE score provides insights into inferred dynamic shifts in CCC potential rather than direct evidence of functional protein interactions, which would require validation through experimental approaches, including proteomics, functional assays, and physiological studies.

### Aggregation of SE scores at the organ level

For each pairwise communication inference type $t_{A↔B}$ (e.g., $t_{\text{Fibroblast}↔\text{Cardiomyocyte}}$, $t_{\text{Endothelial}↔\text{Endothelial}}$) the SE score, ${SE}_{{\text{t}}_{\text{A}↔\text{B}}}$, was computed between two biological states as described above. Communication inference types were then classified based on their SE score: expanding if ${SE}_{t}>\text{0}$, shrinking if ${SE}_{t}<\text{0}$, and stable if $SE_{t}=\text{0}$. Only communication inference types with a net directional change (i.e., those with non-zero SE scores) were included in the subsequent organ-level calculation.

Let $N_{\text{expand}}$ and $N_{\text{shrink}}$ denote the number of communication inference types with positive and negative SE scores, respectively, and let $N_{\text{total}}=N_{\text{expand}}+N_{\text{shrink}}$. The aggregated organ-level SE score ${SE}_{\text{organ}}$, was defined as the normalised difference between expanding and shrinking interactions:


$$SE_{\text{organ}}=\frac{N_{\text{expand}}-N_{\text{shrink}}}{N_{\text{total}}}$$

This metric is bounded between –1 and 1 and reflects the net balance of communication changes at the organ level. Positive values (${SE}_{\text{organ}}>\text{0}$) indicate a net expansion of intercellular communication, negative values (${SE}_{\text{organ}}<\text{0}$) indicate net shrinkage, and a score of zero indicates balanced gains and losses. This aggregated measure enables robust and interpretable comparisons of organ-wide communication dynamics across age, condition, or perturbation, even when the total number of communication inference types varies between tissues.

### Quantitative classification of CCC inference units and their dynamics (the consensus, the gain, and the potential loss)

Building upon the SE score as a quantitative measure of predicted communication changes between biological states, we define ‘CCC inference units’ corresponding to each communication inference type paired with a specific predicted LR interaction. Each CCC inference unit represents a predicted communication event between two cell types, based on transcriptome-inferred LR pairs. We further classify these units into three distinct categories: Consensus (C), Gain (G), and Potential Loss (PL). This framework provides a structured way to infer the predicted stability and dynamics of intercellular communication, enabling systematic identification of interactions that may be conserved or state-specific.

#### Consensus (C).

Consensus CCC inference units represent communication inference types and LR pairs present in both consecutive stages, indicating predicted that interactions persist in presence (though their expression levels may change). For a given communication inference type $t_{A↔B}$ between biological states 1 and 2, the consensus set, $C_{t_{A↔B}}$, corresponds is equal to the intersection set $I_{t_{A↔B}}$ used in the SE score calculation:


$$C_{t_{A↔B}}=I_{t_{A↔B}}\;=L_{t_{A↔B}}^{\left(\text{1}\right)}\cap L_{t_{A↔B}}^{\left(\text{2}\right)}$$

#### Gain (G).

Gain CCC inference units represent communication inference types and predicted LR pairs that newly appear in the comparator state (state 2) but were absent in the reference state (state 1), indicating putative emergent interactions. For a given communication inference type $t_{A↔B}$, the Gain set $\;G_{t_{A↔B}}\;$contains LR pairs present in state 2 but not in state 1. Formally, this is defined as the set difference between state 2 and state 1 transcriptome-inferred LR pairs:


$$G_{t_{A↔B}}=L_{t_{A↔B}}^{\left(\text{2}\right)}\backslash L_{t_{A↔B}}^{\left(\text{1}\right)}$$

Equivalently, the Gain set can be expressed as the set difference between state 2 and the Consensus set $C_{t_{A↔B}}$.

Note that gains represent CCC inference units statistically significant in the comparator state but not predicted as significant in the reference state. However, due to limitations in statistical power and sampling, absence cannot be definitively confirmed, and thus gains should be interpreted as putative emergences of communication rather than confirmed new interactions.

#### Potential Loss (PL).

Potential Loss CCC inference units represent communication inference types and LR pairs present in the reference state (state 1) but absent in the comparator state (state 2), indicating putative loss of interactions. For a given communication inference type $t_{A↔B}$, the Potential Loss set ${PL}_{t_{A↔B}}$, contains LR pairs present in state 1 but not in state 2. Formally, this is defined as the set difference between state 1 and state 2 transcriptome-inferred LR pairs:


$${PL}_{t_{A↔B}}=L_{t_{A↔B}}^{\left(\text{1}\right)}\backslash L_{t_{A↔B}}^{\left(\text{2}\right)}$$

Equivalently, the Potential Loss set can be expressed as the set difference between state 1 and the Consensus set $C_{t_{A↔B}}$.

We term these ‘potential losses’ because while CCC inference tools robustly confirm presence via p-value thresholds, they do not statistically verify true absence. Consequently, CCC inference units not meeting the significance threshold in the comparator state cannot be definitively deemed absent and are therefore classified conservatively as putative losses.

### Statistical limitation on interpreting gains and losses

CCC inference tools identify putative LR interactions based on statistical significance (e.g., p-value thresholds), providing evidence consistent with their presence in a given state. However, these tools do not perform equivalent statistical tests to confirm true absence of interactions. Therefore, both Gains (newly predicted interactions) and Potential Losses (no longer predicted interactions) rely on the presence or absence of statistically significant predictions, which may be influenced by technical variability, sampling depth, or biological noise rather than true biological emergence or disappearance. Consequently, Gains and Potential Losses should be interpreted as predicted, not definitive, changes in intercellular communication potential, acknowledging the inherent limitations of RNA-seq in establishing protein-level activity and the statistical inference framework in confirming interaction potential presence or absence across biological states.

### Quantifying CCC inference unit shifts: Gain and potential loss ratios

To further quantify the dynamics of CCC inference units classified as Gain and Potential Loss, we calculated two complementary metrics for each communication inference type $t_{A↔B}$. The gain ratio, $GR_{t_{A↔B}}$, is defined as the number of newly predicted LR pairs in the comparator state (the Gain set) normalised by the total number of CCC inference units identified in that state:


$${GR}_{t_{A↔B}}=\frac{\left|G_{t_{A↔B}}\right|}{\left|L_{t_{A↔B}}^{\left(\text{2}\right)}\right|}$$

Similarly, the Potential Loss Ratio, $PLR_{t_{A↔B}}$, is calculated as the number of putatively lost LR pairs (the Potential Loss set) normalised by the total CCC inference units identified in the reference state:


$${PLR}_{t_{A↔B}}=\frac{\left|{PL}_{t_{A↔B}}\right|}{\left|L_{t_{A↔B}}^{\left(\text{1}\right)}\right|}$$

By normalising gains and losses relative to the size of their respective stages, these ratios capture the predicted proportion of interactions changing within each communication network, enabling comparison of potential CCC remodelling across different biological contexts, tissues, or experimental conditions, even when total interaction counts differ.

### Statistical analysis of CCC changes

Changes in consensus for each communication inference type were assessed using the Wilcoxon signed-rank test, a non-parametric approach well-suited for paired data without normality assumptions [32]. Given that each LR pair represents a unique, categorical communication unit, the distribution of changes across pairs does not necessarily conform to parametric models. The Wilcoxon test thus provides a robust and appropriate method to evaluate whether observed shifts in the ‘lr_mean’ metric significantly differ from zero across consecutive stages.

The ‘lr_mean’ metric, obtained from the LIANA framework [14], quantifies the average expression level of ligand and receptor genes across interacting cell types and is derived directly from single-cell RNA sequencing data. Although LIANA integrates various inference algorithms with differing scoring strategies, the lr_mean is computed independently and appended as a measure of interaction magnitude, serving as the primary quantitative variable for statistical testing.

A conventional p-value threshold of 0.05 was applied to define significance, reflecting widely accepted statistical practice. While the large scale of the *Tabula Muris Senis* dataset provides substantial power to predict subtle changes in LR interactions, the biological complexity, dependencies among communication pairs, and multiple testing burden require that these p-values be interpreted within a broader biological and statistical context [33]. This considered approach balances statistical rigor with the need for meaningful biological discovery. To further characterize directional shifts in communication, complementary gain ratio (GR) and potential loss ratio (PLR) metrics were calculated to quantify expansions and contractions in LR interactions across stages.

### Molecule shortlisting

Molecule shortlisting was performed to focus the analysis on the top-ranked LR interactions according to the magnitudes of lr_mean, ensuring that only the most relevant molecules were considered for further investigation. CCC inference units were categorised into three groups: consensus, gain, and potential loss, as described above. Each category was prioritised using different metrics to capture meaningful changes in LR interactions across stages. This tailored approach assessed gain and potential loss groups based on the magnitude of LR interaction (lr_mean), while the consensus group was prioritised based on dynamic rank changes of molecules across stages.

For the gain and potential loss categories, shortlisting was based on the lr_mean metric. The K-nearest neighbours (KNN) algorithm (scikit-learn v1.3.2; [34]) was applied to the lr_mean values of CCC inference units within these categories, using a preset two-cluster condition (k = 2). The cluster with the higher average lr_mean was selected to define the shortlisted molecules, reflecting interactions with greater expression magnitude.

In contrast, for the consensus category, shortlisting focused on changes in the relative rank of CCC inference units rather than their absolute lr_mean values. Absolute values of relative rank changes, derived from lr_mean rankings between stages, were computed and then subjected to KNN clustering as performed for gain and potential loss. Molecules exhibiting the highest absolute rank changes were shortlisted as candidates representing consistent but dynamically ranked interactions.

### Comprehensive molecule analysis and frequency-based selection

In the analysis of endothelial cell-to-endothelial cell communication, we first combined all shortlisted ligand–receptor pairs exhibiting notable changes across multiple stages. This approach enabled the identification of molecules consistently altered over time, thereby capturing stable communication dynamics.

For organ-level analysis, ligand–receptor pairs served as the fundamental unit. Each pair was assessed based on its frequency of occurrence within each category and stage. Frequency was calculated by counting the number of distinct communication inference types in which a specific ligand–receptor pair exhibited the same directional change. For example, if the pair FBLN1–ITGB1 showed increased interaction (‘gain’) in both fibroblast-to-fibroblast and cardiomyocyte-to-fibroblast communications between the 1-month and 3-month stages, its frequency in the gain category for that interval would be recorded as two.

This frequency-based approach prioritised ligand–receptor pairs that were recurrent across multiple communication contexts and stages. By selecting the top 20 pairs with the highest frequency of consistent changes, we focused on interactions central to the observed cellular communication patterns, expected to yield the most robust and biologically relevant insights.

These top pairs were then aggregated into final graphs. To further simplify interpretation, we transformed the base unit from ligand–receptor pairs to individual molecules (ligands or receptors), generating single-molecule organ-level graphs that highlight the roles of individual molecules within the communication landscape.

### Gene enrichment analysis

Gene enrichment analysis was performed using the Python package gseapy (version 0.9.5; https://gseapy.readthedocs.io/en/latest/introduction.html; [35]). The ‘GO_Biological_Process_2023’ gene set database was selected to identify relevant signalling pathways and biological processes. To prioritise the most relevant terms, results were ranked based on the odds ratio provided by gseapy. Only the top 20 terms with the highest odds ratios were included in the final visualisations.

### Non-stochastic age pattern permutation test

To test whether ageing affects CCC potential randomly, we hypothesised that, under a stochastic model, the three categories of change (gain, potential loss, or consensus) would be roughly evenly distributed for each ligand–receptor pair. Conversely, if most pairs showed changes in only one category, the null hypothesis would be rejected, indicating that ageing impacts CCC potential in a non-random manner.

For each stage, we identified the 20 most frequent ligand–receptor pairs across all categorical changes. In the permutation simulation, the same number of ligand–receptor pairs was randomly selected from the total set, and categorical change labels were randomly assigned to 20 pairs. The number of overlapping labels was then calculated. This bootstrapping procedure was repeated 200 times to generate a null distribution of overlapping labels.

## Results

### Overview of cell populations and data quality

**Fig 2:**
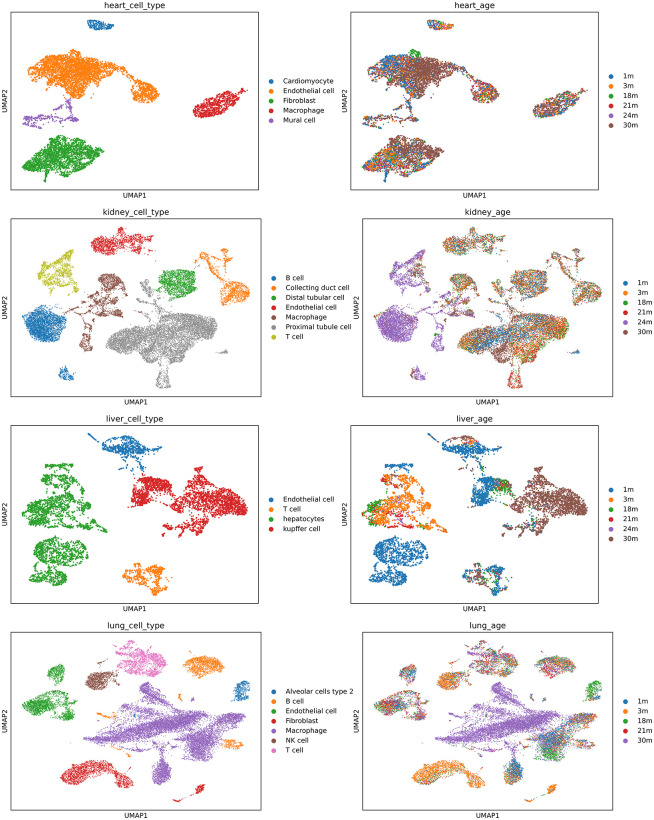
Single cell annotated UMAP projections, labelled by cell type (left) and time point (right). **(A)**: Heart **(B)**: Kidney **(C)**: Liver **(D)**: Lung.

Based on our analysis of the TMS dataset, each organ exhibited a complex and diverse array of cell populations (Fig 2). The 24-month time point was excluded due to poor cell population distribution, particularly in the kidney, which mainly contained B cells and macrophages, lacking other cell types (Fig 2B, Table 2 in S1 Table). Both the kidney and lung data identified seven distinct cell types, which demonstrated a greater variety of cell-cell communication patterns, with different source (signalling) and target (receiving) cell type combinations, compared to the liver.

### Global patterns of putative CCC expansion and contraction across the lifespan

**Fig 3:**
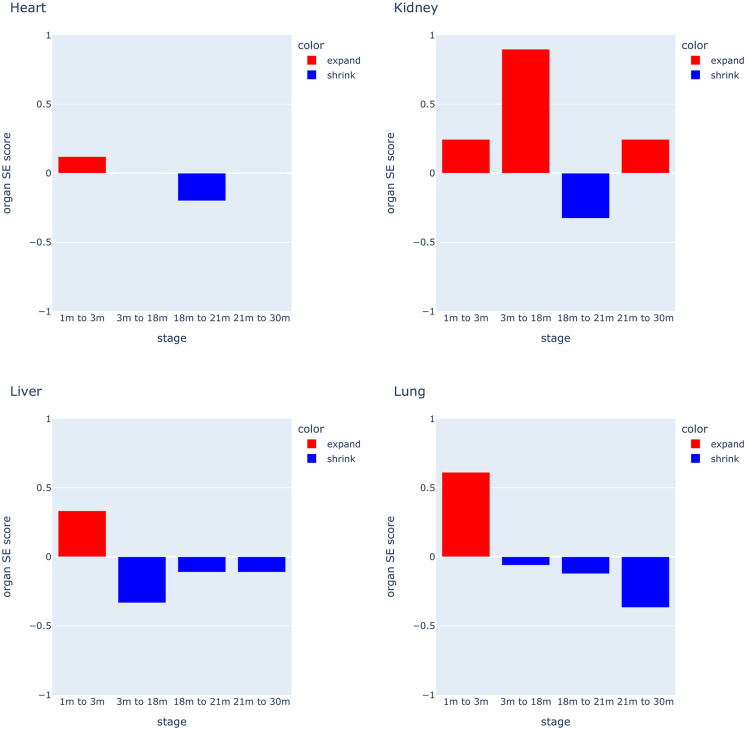
Shrink and expand (SE) score pattern. SE score of 4 organs in different time points to show how CCC shrinks or expands over time, the y-axis indicates the SE score, and the x-axis shows the different time points.

We initially examined the scale of changes in putative CCC across different developmental stages (Fig 3). Both the lung and liver exhibited an expanded CCC potential during the postnatal development phase (1–3 months), followed by a continuous reduction in the predicted level of CCC thereafter (Fig 3). Conversely, the heart maintained a remarkably stable level of CCC potential throughout all time-frames examined. The kidney displayed notable expansion in predicted CCC between 3 and 18 months, achieving the highest SE score of 0.898. Furthermore, we pulled out patterns of putative CCC scale change in types of communication (Fig 1 in S1 Fig) which generated the organ SE scores, categorising them by expansion, contraction, or no change. No single type of communication exhibited a consistent expanding or shrinking pattern across the age-range in each organ.

### Cell type-specific and categorical shifts in predicted communication dynamics

**Fig 4:**
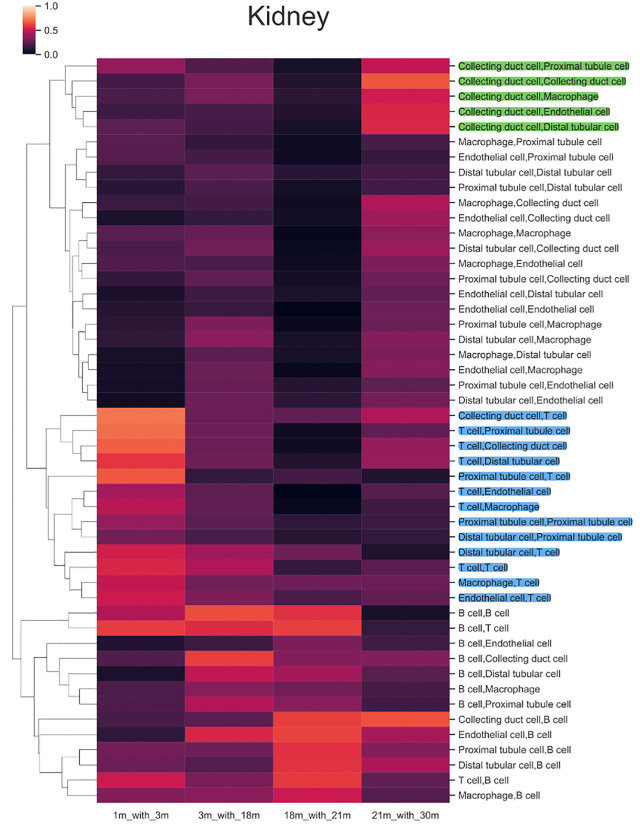
Kidney gain ratios heatmap reflecting the ratio of newly generated CCC inference units. The colour represents the value of the gain ratio: 1 indicates the communication content was completely new in the next time point, and 0 means no new communication content formed by the time point. Y axis: types of communication, X axis: time points. The green and blue colours highlight the T cell and collecting duct cell types of communication discussed in the main text.

Gain ratios, a metric reflecting the proportion of newly predicted CCC inference units, were generated (see methods). In the kidney, ‘B cell-B cell’ and ‘B cell-T cell’ communication inference types maintained high gain ratios, except between 21 and 30 months (Fig 4). T cell related types of communication had gain ratio levels that were notably enhanced between 1 and 3 months across the age-range (Fig 4, blue coloured group), and predicted CCC involving collecting duct cells showed high gain ratio in the aged mouse (21–30 months) (Fig 4, green coloured group). In contrast, the lung displayed a general decrease in putative CCC gain ratio with age, while the liver showed high gain ratio in old age (Fig 2 in S1 Fig). Although the heart did not show a consistent pattern (Fig 2 in S1 Fig), the mural cell related types of communication had gain ratio spikes between 3 and 18 months.

**Fig 5:**
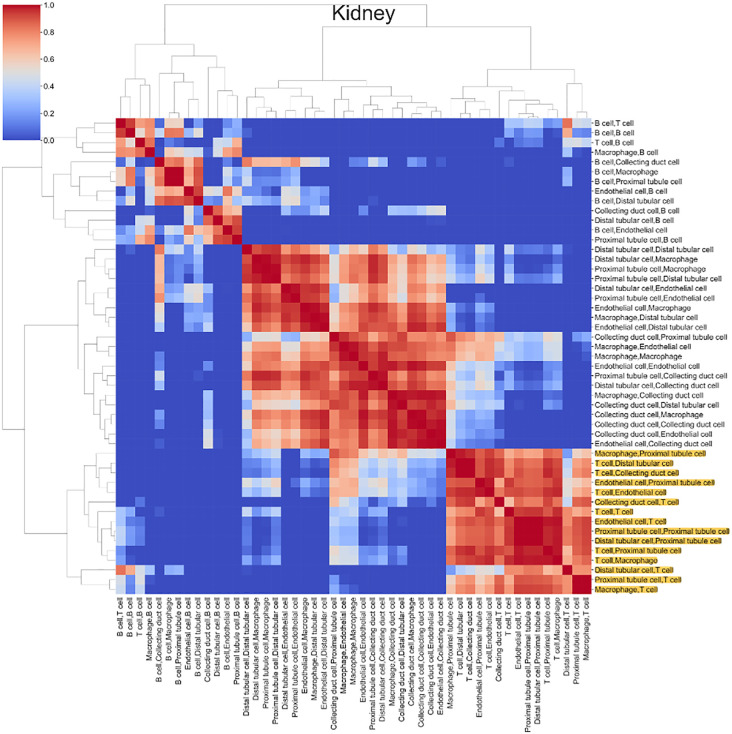
Kidney correlation heatmap of the gain ratio in Fig 4 representing types of communication had the same trend for the new communication generation. There were roughly two clusters of types of communication that had obvious correlations. The T cell correlation cluster was coloured in yellow.

We next clustered the gain ratio vector for CCC potential for each cell-type to discover whether any potential regulatory patterns existed across organs (Fig 5, Fig 3 in S1 Fig). In the kidney, we identified two distinct clusters of B cells and T cells, which were not found in the other organs (Fig 5, Fig 3 in S1 Fig). Additionally, the T cell population appeared to consist of regulatory T cells, as identified by marker genes CD52 and RAC2 (Fig 5, Table 3 in S1 Table) [36].

**Fig 6:**
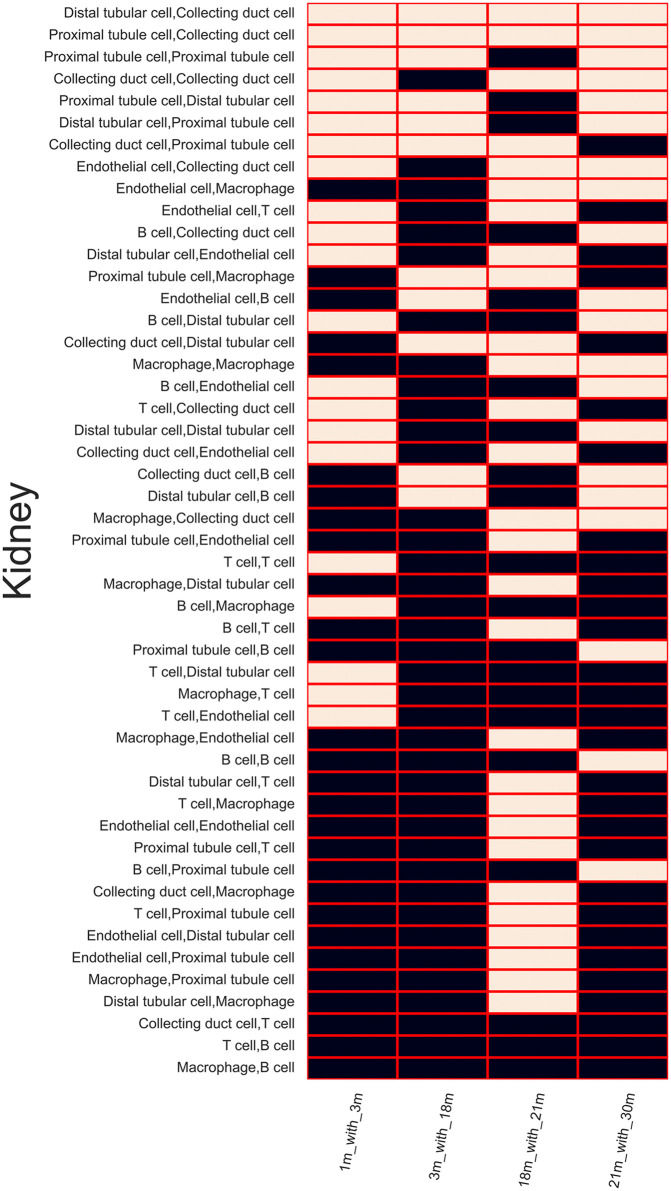
Kidney consensus change binary pattern, white blocks indicate there is a significant change in the shared ligand-receptor pairs between two consecutive ages while the black blocks indicate no significant changes.

Subsequently, we deployed the Wilcoxon signed-rank test to determine whether significant changes occurred between each age stage and the predicted consensus CCC inference units. In the kidney (Fig 6), only predicted CCC types of distal tubular cell-collecting duct cell and proximal tubule cell-collecting duct cell showed consistent consensus changes. Most predicted CCC interactions, however, did not exhibit a consistent, linear progression over time (Fig 6, Fig 4 in S1 Fig), suggesting a complex, non-linear pattern of CCC inference dynamics during ageing.

**Fig 7:**
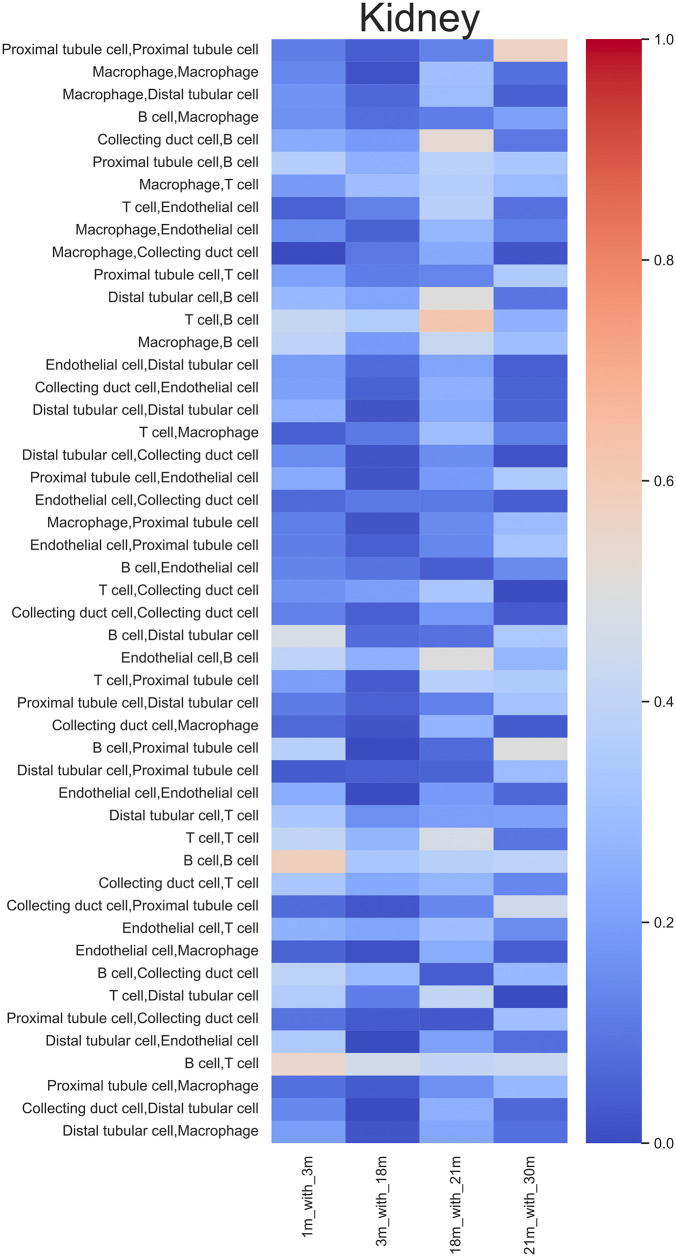
Kidney potential loss ratios heatmap reflecting the ratio of potential lost CCC inference units, the color represents the value of the potential loss ratio, 1 represents that communication content was completely lost in the previous time point, and 0 means all communication in the previous time point was preserved in the next time point.

Furthermore, no consistent patterns of potentially lost CCC inference units were observed across organs or the age range (Fig 7, Fig 5 in S1 Fig). However, several types of CCC potential had relatively high potential loss ratios across organs, exemplified by hepatocyte interactions in the liver (Fig 5 in S1 Fig).

### CCC potential at the molecular level

To uncover molecular associations and linked signalling pathways, we systemically shortlisted molecules and LR pairs for each type of CCC inference unit and organ across gain, potential loss, and consensus categories utilising the ‘lr_mean’ parameter (see methods). This resulted in a comprehensive dataset detailing each organ, each CCC inference unit type, age stage, and categorical changes.

**Fig 8:**
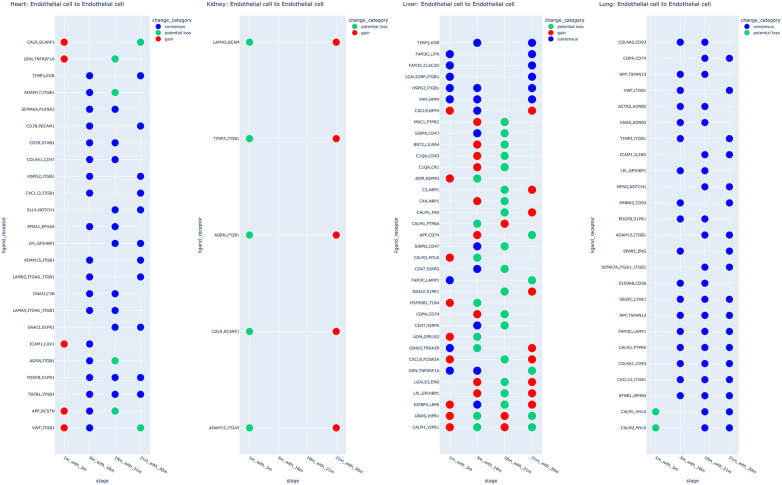
Endothelial cell to Endothelial cell interaction changes in all organs. Ligand-receptors that were shortlisted and had more than one time point change were represented as the key pairs. Colours: change categories, Y-axis: ligand-receptor pairs, X-axis: time points.

Distinct endothelial cell (EC) populations were present across all organs, so we focused on CCC potential occurring between ECs (EC-EC CCC) and selected the LR pairs that had changes over multiple age stages. This was considered for each organ regardless of the change category as shown in (Fig 8). In the kidney, EC-EC CCC potential exhibited minimal changes compared to other organs (Fig 8), with all pairs initially lost regaining presence at old age stage (30 months). This suggests that a simple young versus old comparison does not capture the full range of predicted CCC changes across the lifespan. Furthermore, no significant differences in communication strength (lr_mean) were observed between 1 and 30 months, further highlighting the limitations of simpler early-late comparisons.

In the lung and the heart, EC-EC CCC potential predominantly exhibited a consensus-dominant pattern (>50%) (Fig 8), indicating that predicted EC-EC communication tends to persist across the age range in these organs. By contrast, EC-EC communication inference in the liver showed a mixed pattern comprising all three categorical changes. Furthermore, we aggregated all shortlisted EC-EC change predictions (including those not in Fig 8) across the four organs. It was found that 50 out of 131 predicted LR pairs had changes in more than one organ after which a gene-enrichment analysis was conducted (Fig 6 in S1 Fig). The top term Cardiac Atrium Development (GO:0003230) was generated by three genes (Delta Like Canonical Notch Ligand 4 (DLL4); Notch Receptor 1 (NOTCH1); Endoglin (ENG)).

#### High-frequency CCC pairs and molecules.

**Fig 9:**
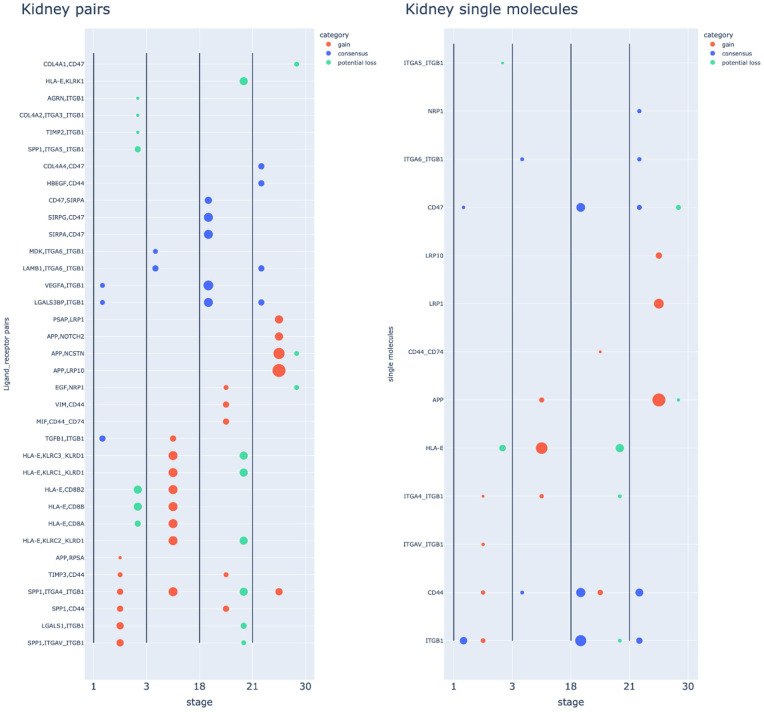
Organ-level molecule analysis of the kidney. **(A)**: high-frequency ligand-receptor pairs, x-axis: time points with different categories of changes, y-axis: ligand (left) to receptor (right), colours show categories of changes, and the size of the dot indicates the frequency of changes in the organ (the number of types of communication for each particular pair). Most changes of pair within a single time point did not possess more than one categorical change. The pairs shown here are a subset of the top 20 pairs (see methods) for ease of visualisation, with all shown in Fig 7 in S1 Fig. **(B)**: high-frequency single molecule patterns for the top 20 single molecules selected based on the frequency within each time point on each categorical change. The single molecules shown here are a subset of top 20 single molecules (see methods), with all the top 20 single molecules in Fig 8 in S1 Fig.

In our final analysis, we shortlisted putative LR pairs from all types of CCC potential within each stage across the age range. Subsequently, we sorted these pairs and selected the top 20 pairs based on frequency. Frequency reflects how many different types of communication potential (i.e., predicted cell-type and LR pairs) had the exact categorical change at a given LR pair, with higher frequency suggesting a more central role in intercellular signalling within the organ (see methods). The outcomes from all three categories were again compiled for each organ. As shown in Fig 9A and Fig 7 in S1 Fig, within one stage of the age range, most predicted pairs did not have multiple categorical changes, instead, a single categorical change was most common. We compared the predicted number of overlapping LR pairs in each stage and organ using a permutation analysis (see methods). In all cases, overlap values were lower than the p = 0.05 cutoffs of the simulated distributions, indicating that the effects of ageing on CCC inference unit categorical shifts appear ordered and highly non-stochastic (Table 4 in S1 Table). This pattern suggests that the impact of age on CCC potential is not stochastic in nature. In addition, most of the CCC related pairs also did not show consistent changes across the time range but were quite specific to one or two age stages with no obvious linear change across the range.

Recognising that drug targeting typically focuses on single molecules rather than LR pairs, we next derived a molecular-level ranking by counting occurrences of predicted individual molecules within the top LR pairs for each organ (Fig 9B). At the single-molecule level, we observed a complex pattern in which many molecules have multi-categorical and continuous changes across all stages and all organs. This is predicted for molecules such as CD44 antigen (CD44), Integrin Subunit Beta 1 (ITGB1), and Low Density Lipoprotein Receptor-Related Protein 1 (LRP1) (Fig 9B, Fig 8 in S1 Fig). GE analysis was performed on high-frequency pairs (Fig 9 in S1 Fig) and a consistent presence of the term “Astrocyte Activation” was found in each organ, as well as “Positive Regulation Of Amyloid-Beta Clearance” in three organs. The predicted gene sets generating the ”Astrocyte Activation” term were different between organs but had many overlapping genes, including Amyloid-Beta Precursor Protein (APP), Complement C1q A Chain (C1QA), Granulin Precursor (GRN), LRP1, Triggering Receptor Expressed on Myeloid Cells 2 (TREM2), and Complement Component 5a Receptor 1 (C5AR1) (Table 5 in S1 Table).

## Discussion

In this study, we developed a hierarchical analytical framework to examine putative CCC dynamics across ageing, a strategy that is generalisable to arbitrary comparisons of biological state, including developmental, disease-related, or treatment-induced changes. CCC potential was structured into three levels (predicted LR pairs, communication inference types (cell–cell pairs), and organ context) and directional changes between states, defined by the predicted presence or absence of interactions, were quantified using the Shrink–Expand (SE) score. Building on the SE score, individual CCC inference units were classified as Gain, Consensus, or Potential Loss, enabling fine-grained, interpretable analysis of signalling changes across time.

To quantify global shifts in CCC potential, we introduced the Shrink–Expand (SE) score, a normalised metric ranging from –1–1 that captures the net balance of gained and lost interactions while accounting for their overlap between timepoints. The SE score can be calculated at the communication-type level and aggregated to provide a robust summary of predicted organ-wide CCC remodelling. Unlike simple edge-count metrics [37], our approach provides a more nuanced insight into the dynamics of CCC inference across complex tissues.

We stratified cells by annotated type to interpret predicted intercellular communication patterns. However, some predicted interactions may also occur within cells of the same type (intra-typical) or reflect broader shared signalling programs across the ensemble of cells. Accordingly, identified gains, maintenance, or losses of interactions across the lifespan capture changes in the repertoire of predicted intercellular communication potential, without implying direct mechanistic validation for any individual cell-to-cell interaction.

Applying this approach to four major organs across the murine lifespan (1–30 months, with >24 months considered aged [38]), we observed a consistent expansion of CCC potential during early postnatal development (1–3 months), particularly notable in the kidney. While basic kidney function is largely established by 14 days after birth [39], this predicted expansion in CCC potential likely reflects the ongoing refinement and stabilisation of predicted intercellular signalling networks following functional maturation, rather than a predicted acquisition of additional organ functionality. At the other extreme, 30 months corresponds to advanced ageing under laboratory conditions and may capture late-life processes not representative of typical ageing trajectories [38]. Our multi-stage, longitudinal analysis mitigates potential overinterpretation of the influence of any singular time point when assessing the tapestry of putative communication signatures by comparing consecutive stages across the full lifespan rather than relying solely on early–late dichotomies.

However, we note that our analyses rely on static measurements at discrete intervals. Consequently, while the SE framework captures gains, consensus, or potential loss of predicted CCC interactions between these defined intervals, we cannot infer what may have been predicted at intermediate ages not sampled. Thus, changes within intervals could have transiently emerged or resolved, and differences should be interpreted as interval-specific summaries rather than continuous trajectories of CCC potential.

While the SE score provides a normalised and interpretable metric for quantifying the scale of shifts in CCC potential, it may be influenced by the relative abundance of cell types and the sensitivity of LR inference and prediction. Rare or lowly-expressed putative communication events might be underrepresented, and the SE score implicitly assumes that gained and lost interactions could plausibly contribute equally to biological impact, which may not always hold true. Future refinements could incorporate weighting schemes based on functional relevance to the desired subclass of communication potential or incorporate protein-level data to further improve robustness.

We also recognise that microRNA regulation, mRNA half-life variability, and post-transcriptional modifications can contribute to discrepancies between the mRNA detected in single-cell data and the proteins actually present or active [40]. These factors represent key limitations of transcriptome-based CCC inference and highlight the importance of integrating multi-omics datasets in future studies to more reliably link predicted transcriptomic changes to protein expression and functional intercellular signalling outcomes. In this manner, our framework could ultimately move from mapping comparative communication potential to identifying validated fluctuations in communication events.

After categorizing CCC inference units across different stages of the lifespan, we first focussed on gained interactions, those that emerged at subsequent time points. In the kidney, T cell communication stood out, particularly a subset marked by high expression of CD52 and RAC2. Previous studies have suggested that CD52 + T cells represent a regulatory T cell population, possibly derived from inducible or adaptive Tregs [41]. Clinically, CD52 is a known therapeutic target in diseases such as chronic lymphocytic leukaemia and multiple sclerosis [42,43], and its levels have been shown to influence kidney transplant outcomes [44–46]. These findings, together with our observed clustering of T cell CCC inference units in the kidney, support the hypothesis that CD52 + T cells may play a central role in maintaining or regulating kidney-specific intercellular signalling across ageing. In contrast, the B cell cluster, while exhibiting a weaker correlation pattern, consisted exclusively of B cell-associated CCC inference units. This may suggest a functionally distinct, independent role for B cell-mediated CCC in kidney ageing.

In the heart the most prominent predicted age-specific gain was observed in the mural cell related types of CCC potential at the 3–18 months interval. Prior studies have shown that LYVE-1 + macrophages can regulate vascular tone through interaction with mural cells [47]. Our observation of a gain ratio spike in these types of communication points to a potentially underexplored regulatory axis of mural cells in cardiovascular ageing. Across organs, we did not observe such strong or consistent CCC inference unit clusters except kidney, suggesting that these types of communication are likely largely independent from each other’s influence during ageing.

Few CCC inference units showed consistent consensus-level changes over time, and the overall potential loss ratio was low across all organs. This indicates that once CCC is inferred to be established, CCC potential tends to remain stable, with relatively few interactions becoming entirely lost. Such persistence in CCC potential could reflect the maintenance of key homeostatic or functional pathways.

At the molecular level, changes in CCC potential across ageing were found to be highly heterogeneous, both within and between organs. Focusing on a specific CCC inference type, endothelial-to-endothelial (EC–EC) interactions, we predicted that the same LR pairs fluctuated in presence across multiple intermediate stages in the kidney, even if they appeared persistent when comparing only the earliest (1 month) and latest (30 months) time points. These predictions suggest that simple early–late comparisons can obscure transient or cyclical dynamics that are detectable only through multi-stage, longitudinal analyses. In the context of CCC potential, such transient fluctuations may reflect critical windows of development, adaptation, or decline that would otherwise be missed. Therefore, our multi-stage framework offers a more nuanced approach and temporally resolved view for capturing the complexity of predicted intercellular signalling patterns over time. We identified a substantial proportion of shared LR pairs predicted to be involved in mediating EC–EC communication across multiple organs, suggesting recurring signalling patterns between endothelial cells. However, despite this overlap, the same pairs often exhibited organ-specific dynamics, changing in direction or at different timing depending on the tissue context.

At the organ level, we found that frequently changing LR pairs tended to cluster within specific age stages and change categories (i.e., gain, consensus, potential loss), further supporting the idea that transcriptome-inferred CCC remodelling is not random or linear, but rather stage-specific and shaped by organ context. Notably, despite the organ-specific differences in CCC inference unit composition, gene set enrichment analysis revealed convergence onto shared signalling pathway terms. For instance, the recurrent enrichment of the “Amyloid-Beta Clearance” term was predicted across all four organs studied. Although amyloid deposits can accumulate pathologically in certain contexts, such as Alzheimer’s disease [48–50], they also occur in tissues of individuals without overt pathology (e.g., restrictive cardiomyopathy [51]). Thus, the observed enrichment of “Amyloid-Beta Clearance” term related to predicted communication changes likely reflects conserved, physiological regulatory programs that operate across organs to maintain homeostasis, rather than being solely indicative of disease processes. These findings suggest that the putative orchestration of CCC potential inferred from transcriptomics could serve as a proxy for coordinated, age-associated tissue maintenance beyond the central nervous system.

When we deconstructed inferred LR pairs into individual molecules, additional complexity emerged that was not apparent at the pairwise level. Given the extensive variation of these molecules across ages, their use as drug targets may require careful consideration, as interventions could have age-dependent effects. This was particularly evident for certain high-frequency molecules such as the CD44 receptor, whose predicted CCC potential was observed across multiple organs and communication inference types, often in an age-specific manner. Recent work has implicated CD44 in the decline of endothelial cell autophagy, a process thought to contribute to age-related vascular dysfunction [52]. Our findings suggest that transcriptomic representation of CD44-related CCC inference pattern shifts across normal ageing may capture aspects of cross-organ regulation associated with normal ageing.

While our study provides a detailed analysis of CCC inference dynamics across ageing and multiple organs, several limitations should be acknowledged. Our analyses rely exclusively on transcriptomics, namely scRNA-seq data, which captures a subset of the total cellular gene expression, serving as an informative proxy for potential intercellular communication, and neither protein abundance, nor spatial proximity, nor signalling activity. Consequently, the inferred LR interactions remain putative hypotheses of communication potential, pending validation through protein-level assays, spatial transcriptomics, proteomics, or combinations thereof. In addition, transcriptomic inference of CCC is inherently limited to peptide-based ligands, thereby overlooking non-peptide mediators such as steroid hormones, lipid-derived signalling and other small molecules or ions [13]. This likely leads to an underestimation of the full spectrum of CCC dynamics. Future integration with proteomic, metabolomic, or spatial multi-omics datasets will be critical to provide a more comprehensive and mechanistically grounded view of intercellular communication.

While certain LR pairs catalogued in curated resources (e.g., GPCR–G protein–protein interaction pairs such as ADRB3–GNAS) are intracellular by canonical definition, their transcriptomic co-expression across distinct annotated cell types may indirectly reflect coordinated signalling programs or shared regulatory networks that could influence intercellular communication potential. We retained these interactions in our analysis to preserve completeness and comparability with reference resources, and to allow the Shrink and Expand (SE) framework to quantify system-level shifts in inferred intercellular communication potential, capturing higher-order patterns of protein–protein interaction co-activity across populations. Retaining these interactions also enables the framework to support hypothesis generation regarding biological-state transitions (e.g., ageing, disease, or treatment responses), by highlighting candidates that may participate in context-dependent shifts in intercellular communication. Importantly, detection by LIANA and analysis via SE score should not be interpreted as direct mechanistic evidence of intercellular cell–cell signalling, and this caveat is emphasized when interpreting our results.

Beyond technical limitations, biological factors such as shifts in cell-type composition with age, sex differences, and environmental influences may also confound CCC inference. While the TMS dataset provides a valuable snapshot across the lifespan, future studies incorporating controlled cohorts or stratified analyses will be necessary to dissect how these variables modulate predicted CCC patterns. Additionally, cell dissociation and isolation procedures may introduce biases affecting the representation of fragile or rare cell types, which could influence downstream communication analyses.

To mitigate batch effects and technical confounders in our cross-stage comparisons, we a consensus-based strategy emphasising reproducible relative changes in inferred patterns of CCC potential rather than direct gene-level differential expression. This approach may prove valuable when applied to complementary ageing datasets, such as the recently published mouse scRNA-seq dataset covering adult stages [53], which, despite its narrower age range, could benefit from our hierarchical framework to predict and extract biologically consistent communication patterns from lifespan-resolved data.

Although our present results are derived from the TMS dataset, the analytical framework itself is broadly generalisable and adaptable to diverse biological contexts, including disease models, tissue regeneration, and pharmacological perturbation. Future integration with emerging multi-omics modalities (e.g., single-cell epigenomics, spatial transcriptomics, functional proteomics) promises to further refine the resolution and interpretability of CCC inference and derived longitudinal dynamics, advancing toward a more mechanistically grounded understanding of intercellular communication across physiological and pathological ageing. In doing so, this and subsequently enhanced may lay the groundwork for discovering of stage- and condition-specific biomarkers and informing targeted therapeutic strategies for age-associated and related diseases.

## Conclusions

In summary, we present a novel, hierarchical computational framework that extensively characterises transcriptome-inferred CCC dynamics across ageing at multiple biological scales, from predicted LR pairs, to potential communication inference types between cell pairs, to organ-level summaries. Applying this framework to the TMS dataset spanning 1–30 months of murine life, we reveal intricate, non-linear, and organ-specific patterns of predicted CCC expansion and remodelling during development, maturation, and ageing, many of which are obscured by binary early-vs-late comparisons.

Our analyses highlight specific cell populations (e.g., CD52 + T cells in the kidney, macrophage–mural cell pairs in the heart) and molecular players (such as CD44) that emerge as potential regulators of age-dependent intercellular communication, as well as convergent signaling pathways such as amyloid-beta clearance across multiple organs, pointing to systemic mechanisms of CCC during ageing. While these observations remain putative and inferred from transcriptomic data, they provide hypotheses for future orthogonal validation using protein-level, spatial, or multi-omics approaches.

Ultimately, this work establishes a powerful computational paradigm for decoding the inferred dynamic landscape of intercellular communication across the lifespan, offering new avenues for understanding ageing biology and guiding hypotheses for therapeutic innovations.

## Supporting information

S1 Fig All Supplementary figures cited in the main text. (DOCX)

S1 Table All Supplementary tables cited in the main text. (XLSX)

S1 Dataset Containing all curated datasets for four organs. (ZIP)
