# Supplementary material for: Quantitative profiling of lifespan-dependent cell-cell communication potential reveals dynamic ligand-receptor network shifts across mouse tissues
Source: PLoS One. 2026 Mar 20;21(3):e0345045. doi: 10.1371/journal.pone.0345045 (PMC13004335; doi:10.1371/journal.pone.0345045)
Supplement: S1 Fig — (DOCX) [file pone.0345045.s001.docx]

Fig1 type of communication SE scores in kidney, heart and lung

The full distribution of individual communication type’s SE score is judged by above 0 or not, left(kidney), middle(heart), right(lung), the y-axis: source(left) cell type to target(right) cell type, red color means the scale change was positive (expanding), blue indicates shrinking.

Fig2, gain ratio pattern in the heart, liver, and lung.


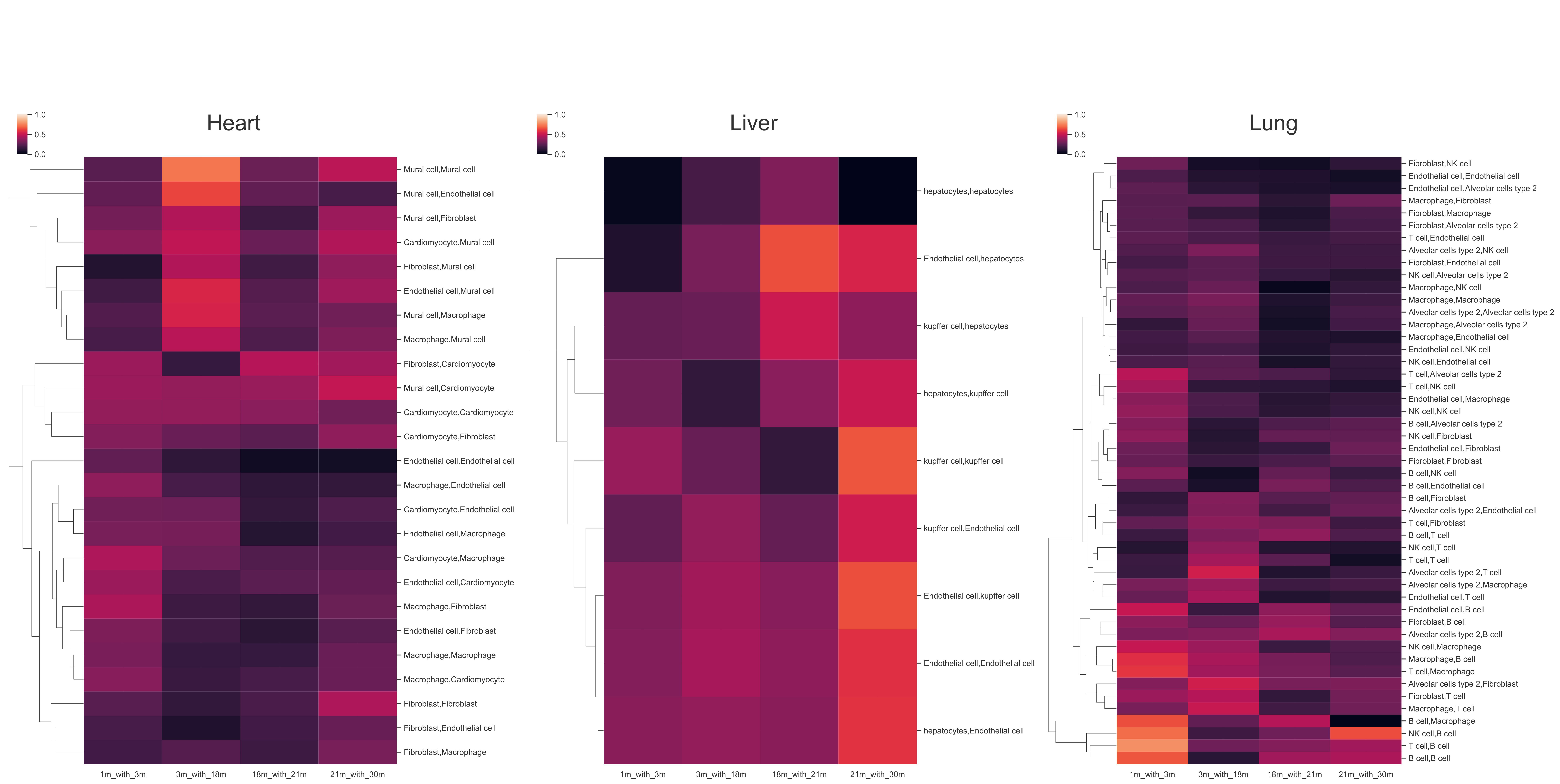


Gain ratios heatmap reflecting the ratio of newly generated CCC units, the color represents the value of the gain ratio. 1 represents the communication content was completely new in the next stage, and 0 means no new communication content formed by the stage. Y axis: types of communication, X axis: stages

Fig3, gain ratio correlation clustering heatmap, from left to right: heart, liver, and lung.





Correlations between types of communication in each organ. Types of communication that had similar gain ratio change patterns were clustered together.

Fig4 signed-rank test on consensus ligand-receptor pairs.


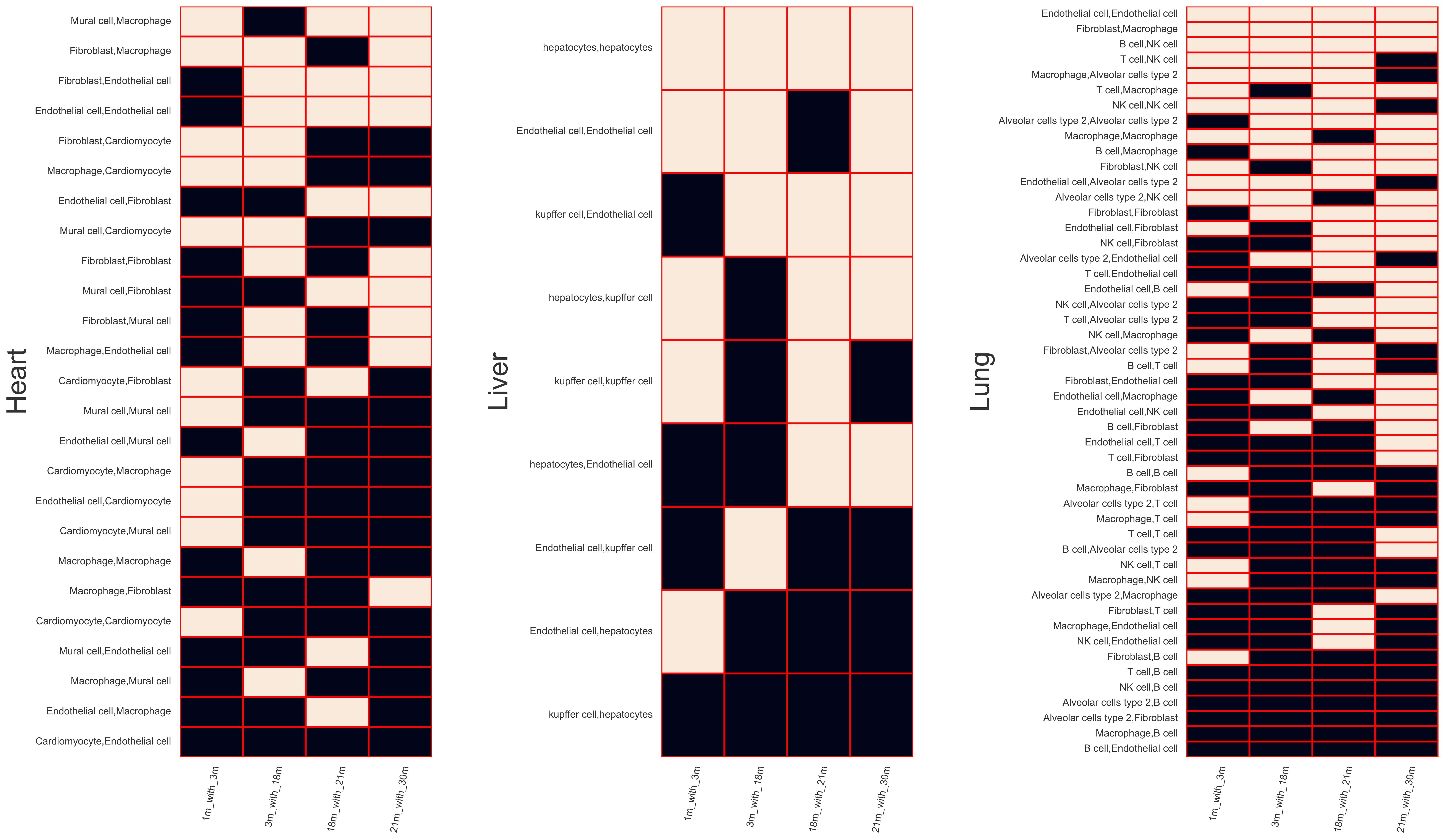


In the consensus change pattern, white blocks indicate there is a significant change in the shared ligand-receptor pair between two consecutive ages while the black blocks mean no significant changes.

Fig5 potential loss pattern


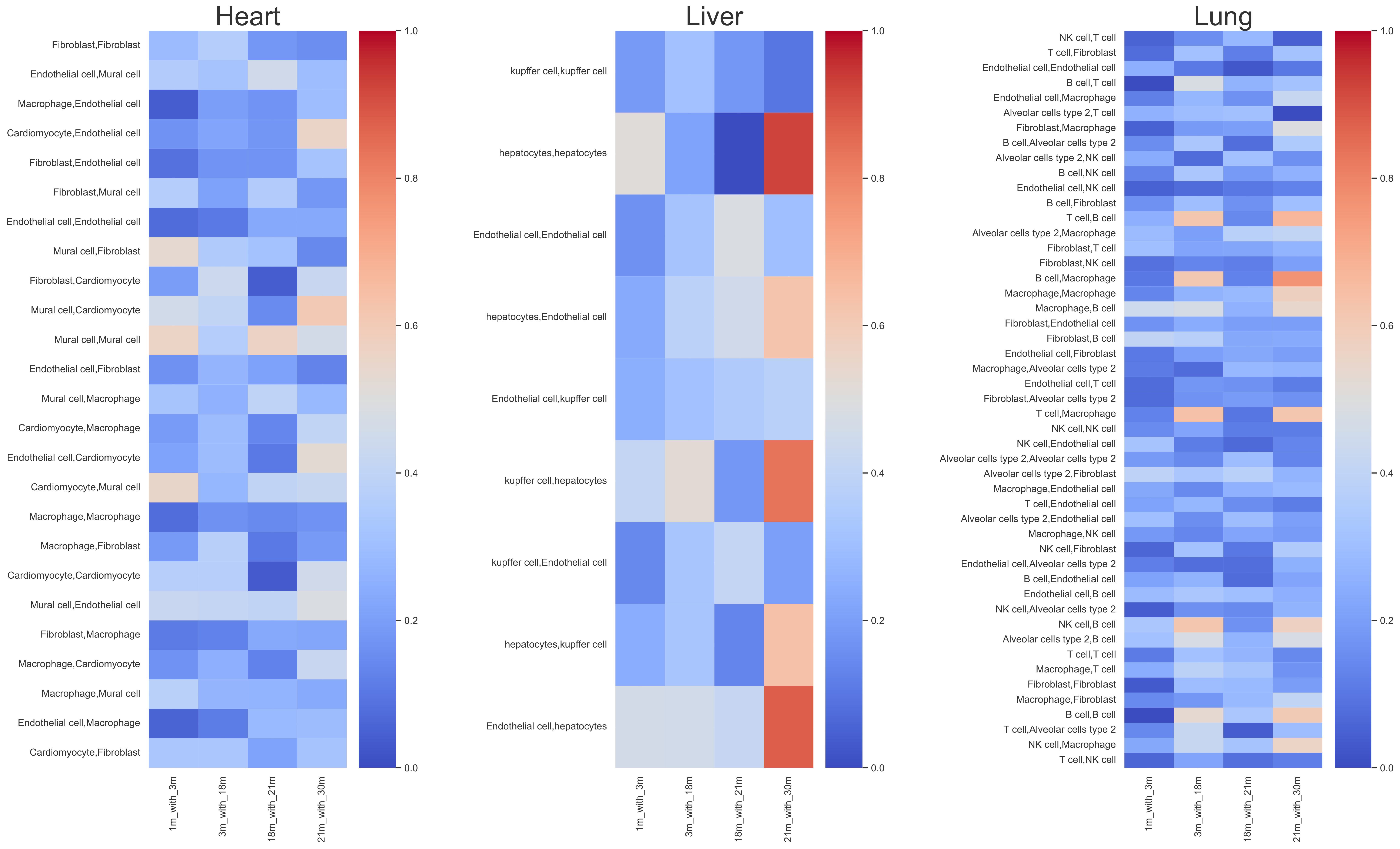


potential loss ratios heatmap reflecting the ratio of potential lost CCC units, the color represents the value of the potential loss ratio, 1 represents that communication content was completely lost in the previous stage, and 0 means all communication in the previous stage was preserved in the next stage.

Fig6 GE term analysis on EC-EC muti organ ligand and receptor

Gene enrichment analysis on multi-organ pairs in main text Fig8. Based on the odds ratio, the top 20 terms were selected. All terms had lower than 0.05 adjusted p values.

Fig7 high frequency ligand receptor pairs at the organ level.





High-frequency ligand-receptor pairs, x-axis: stages with different categories of changes, y-axis: ligand (left) to receptor (right), colors also showed categories of changes, and the size of the dot indicated the frequency of changes in the organ, frequency represented the number of types of communication for each particular pair. Most changes of pair within a single stage did not possess more than categorical change.

Fig8 high frequency single molecules in each organ.





high-frequency single molecules pattern, top 20 single molecules based on the frequency were selected within each stage on each categorical change. X-axis: stages with different categories of changes, y-axis: single molecules, colors also showed categories of changes, and the size of the dot indicated the frequency of changes in the organ, frequency represented the number of types of communication for each particular single molecules.

Fig9 gene enrichment analysis on high-frequency pairs


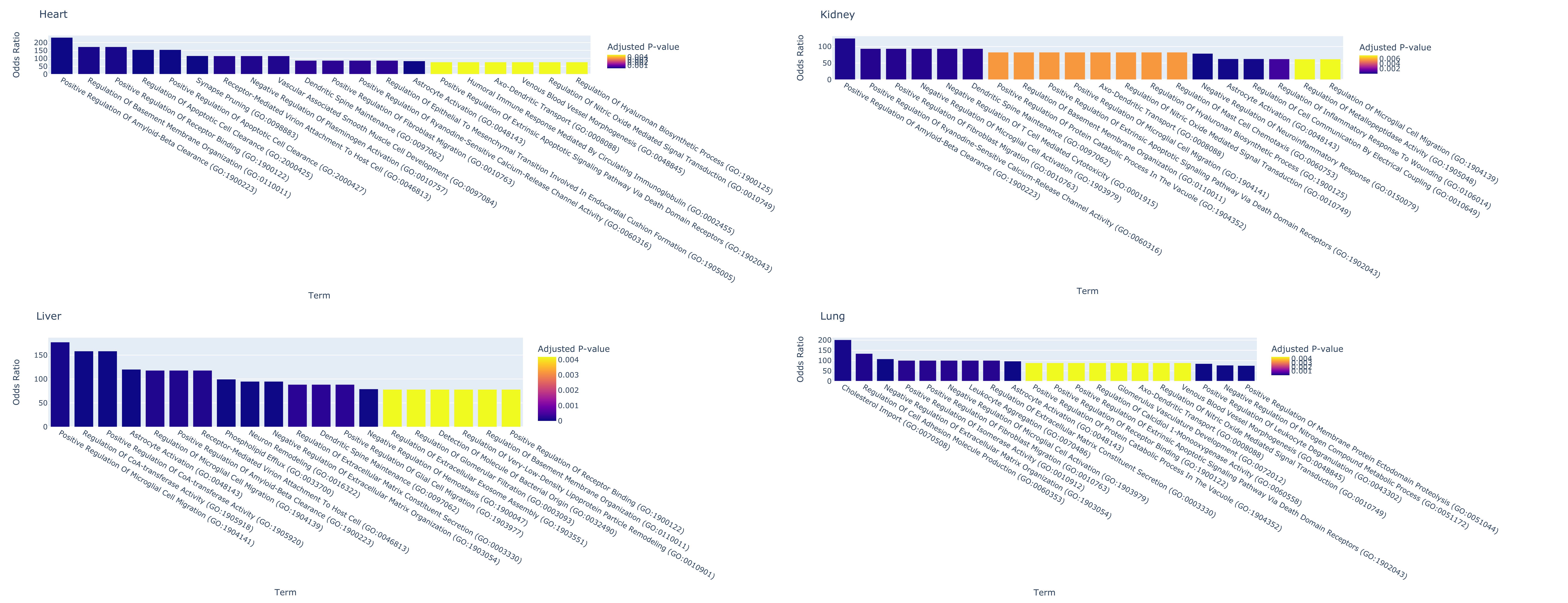


Gene enrichment analysis on high-frequency pairs in sup fig6. Based on the odd ratio, the top 20 terms were selected. All terms had lower than 0.05 adjusted p values.
